# Initial observations on sexual dysfunction as a symptom of chemotherapy-induced peripheral neuropathy

**DOI:** 10.3205/000322

**Published:** 2023-06-23

**Authors:** Nadine Reimer, Dirk Brodesser, Dominik Ratiu, Damir Zubac, Helmar C. Lehmann, Freerk T. Baumann

**Affiliations:** 1 University of Cologne, Department I of Internal Medicine, Center for Integrated Oncology Aachen Bonn Cologne Dusseldorf, University Hospital of Cologne, Germany; 2 University of Cologne, Clinic and Polyclinic for Gynecology and Obstetrics, University Hospital Cologne, Germany; 3 University of Cologne, Clinic and Polyclinic for Neurology, University Hospital Cologne, Germany

**Keywords:** peripheral neuropathy, genital organs, sexual dysfunction, physical activity, oncology, exercise therapy

## Abstract

**Introduction:**

Peripheral neuropathy (PNP) in feet and/or hands and sexual dysfunction are common side effects of cancer therapies. In patients with other diseases, there is evidence of an association between peripheral nervous system disorders and sexual dysfunction due to the impact of impaired neuronal control on genital organ sensitivity. In cancer patient interviews, it has now been observed that PNP and sexual dysfunction may be related. The aim of the study was to investigate the potential association between PNP, sexual dysfunction, and physical activity behavior.

**Methods:**

Ninety-three patients with PNP of the feet and/or hands were interviewed in August/September 2020 in a cross-sectional study regarding medical history, sexual dysfunction and functionality of the genital organs.

**Results:**

Thirty-one persons who participated in the survey provided seventeen evaluable questionnaires (four men, thirteen women). Nine women (69%) and three men (75%) reported sensory disorders of the genital organs. Three men (75%) had erectile dysfunction. All men who had sensory symptoms of the genital organs received chemotherapy, and one man also received immunotherapy. Eight women were sexually active. Five (63%) of them reported genital organ symptoms and mainly lubrication disorders. Four (80%) of the five sexually inactive women reported genital organ symptoms. Eight of the nine women with sensory symptoms of the genital organs received chemotherapy, and one woman received immunotherapy.

**Discussion:**

Our limited data suggest genital organ sensory symptoms in chemotherapy and immunotherapy patients. Genital organ symptoms do not appear to be directly related to sexual dysfunction, and the association between PNP and genital organ symptoms appears to be more pronounced in sexually inactive women. Chemotherapy could cause sensory symptoms of the genital organs and sexual dysfunction by damaging genital organ nerve fibers. Chemotherapy and anti-hormone therapy (AHT) could trigger a disturbance of the hormone balance, which in turn could be causative for sexual dysfunction. It remains open whether the cause of these disorders is the symptomatology of the genital organs or the altered hormone balance. The significance of the results is limited due to the small number of cases. To our knowledge, this study is the first of its kind in cancer patients and allows a better understanding of the association between PNP, sensory symptoms of the genital organs, and sexual dysfunction.

**Conclusion:**

In order to be able to narrow down the cause of these initial observations in cancer patients more precisely, larger studies are needed that can relate the influence of cancer therapy-induced PNP, physical activity level and hormone balance to sensory symptoms of the genital organs and sexual dysfunction. The methodology of further studies should take into account the frequent problem of low response rates in surveys on sexuality.

## Zusammenfassung

**Einleitung:**

Polyneuropathie (PNP) in Füßen und/oder Händen und sexuelle Dysfunktion sind häufige Nebenwirkungen von Krebstherapien. Bei Patient*innen mit anderen Erkrankungen gibt es Hinweise auf einen Zusammenhang zwischen Erkrankungen des peripheren Nervensystems und der sexuellen Dysfunktion aufgrund des Einflusses der gestörten neuronalen Kontrolle auf die Sensibilität der Genitalorgane. In Gesprächen mit Krebspatient*innen wurde nun beobachtet, dass die PNP und die sexuelle Dysfunktion möglicherweise zusammenhängen. Ziel der Studie war, den potentiellen Zusammenhang zwischen PNP, sexueller Dysfunktion und körperlichem Aktivitätsverhalten zu untersuchen.

**Methoden:**

Dreiundneunzig Patient*innen mit PNP der Füße und/oder Hände wurden im August/September 2020 in einer Querschnittuntersuchung bezüglich Krankengeschichte, sexueller Dysfunktion und Funktionalität der Genitalorgane befragt.

**Ergebnisse:**

Einunddreißig Personen, die an der Umfrage teilnahmen, lieferten siebzehn auswertbare Fragebögen (vier Männer, dreizehn Frauen). Neun Frauen (69%) und drei Männer (75%) berichteten über Gefühlsstörungen an den Geschlechtsorganen. Drei Männer (75%) hatten eine erektile Dysfunktion. Alle Männer, die sensorische Beeinträchtigungen der Genitalorgane aufwiesen, erhielten Chemotherapie, und ein Mann zudem Immuntherapie. Acht Frauen waren sexuell aktiv. Fünf (63%) von ihnen berichteten von Symptomen der Genitalorgane und hauptsächlich Lubrikationsstörungen. Vier (80%) der fünf sexuell inaktiven Frauen berichteten von Symptomen der Genitalorgane. Acht der neun Frauen mit sensorischen Symptomen der Genitalorgane erhielten Chemotherapie und eine Frau erhielt Immuntherapie.

**Diskussion:**

Unsere begrenzten Daten deuten auf sensorische Symptome der Genitalorgane bei Chemotherapie- und Immuntherapie-Patient*innen hin. Symptome der Genitalorgane scheinen nicht direkt mit sexueller Dysfunktion zusammenzuhängen und der Zusammenhang zwischen PNP und Symptomen der Genitalorgane scheint bei sexuell inaktiven Frauen ausgeprägter zu sein. Chemotherapie könnte durch Schädigung von Nervenfasern der Genitalorgane sensorische Symptome im Genitalbereich und eine sexuelle Dysfunktion verursachen. Chemotherapie und Anti-Hormontherapie (AHT) könnten eine Störung des Hormonhaushaltes auslösen, die wiederum ursächlich für eine sexuelle Dysfunktion sein könnte. Es bleibt offen, ob eine Symptomatik an den Genitalorganen oder der veränderte Hormonhaushalt Ursache dieser Störungen ist. Die Aussagekraft der Ergebnisse ist aufgrund der geringen Fallzahl eingeschränkt. Diese Untersuchung ist nach unserer Kenntnis die erste dieser Art bei Krebspatient*innen und ermöglicht ein besseres Verständnis für den Zusammenhang von PNP, sensorischen Symptomen der Genitalorgane und sexueller Dysfunktion.

**Fazit:**

Um die Ursache dieser ersten Beobachtungen bei Krebspatient*innen genauer eingrenzen zu können, sind größere Studien notwendig, die den Einfluss der Krebstherapie-induzierten PNP, des körperlichen Aktivitätsniveaus und des Hormonhaushalts auf die sensorischen Symptome der Genitalorgane und die sexuelle Dysfunktion in Beziehung setzen können. Bei der Methodik weiterer Studien sollte das häufig auftretende Problem der geringen Rücklaufquoten in Befragungen zur Sexualität berücksichtigt werden.

## Introduction

Peripheral neuropathy (PNP) occurs in 19–85% of cancer patients due to chemotherapy and immunotherapy. It mostly affects sensory nerve fibers, but may also affect the motor and autonomic nerves [1]. In addition, a very common side effect of cancer therapies is sexual dysfunction, which is multifactorially caused by physiological, psychological, and interpersonal parameters [2], [3], [4]. Physically more active patients have sometimes significantly better sexual function than those with lower or no physical activity [5], [6]. Erectile dysfunction is clinically associated with, for example, endocrinologic, neurogenic, vasculogenic, and systemic diseases such as cardiovascular disease. It is caused by diabetes, hypertension, obesity, or physical inactivity and is also a predictor of coronary heart disease [7]. There is evidence of an association between disorders of the peripheral nervous system, especially peripheral neuropathies of the autonomic nervous system, and sexual dysfunction in amyloidosis, cauda equina lesion, diabetes, or lesion of the pelvic nerve patients. In men and women, the resulting impaired neuronal control may limit genital organ sensitivity, orgasm, erection, ejaculation, lubrication, and, in some cases, libido [8]. 

In our care structure, some patients with proven PNP in feet and/or hands additionally reported symptoms in the genital organs similar to those of PNP in feet and hands. To our knowledge, this symptom has not been reported in cancer patients in the literature, and this could be the first paper about this symptom in cancer patients. The aim was to investigate a potential relationship between PNP, sexual dysfunction, and physical activity behavior in cancer patients. 

## Methods

Medical records of cancer patients that have been treated at the Oncological Training and Exercise Therapy (OTT) of the University Hospital Cologne were retrospectively screened for inclusion criteria of cancer diagnosis, adult age, and proven PNP in feet and/or hands. The next step was to assess whether they also had or have sexual dysfunction and symptoms of the genital organs similar to those of PNP in feet and hands. Therefore, a cross-sectional survey was conducted, and ninety-three PNP patients of the OTT were contacted via online questionnaire in August/September 2020 regarding potential PNP associated sexual dysfunction of the genital organs. The reporting of PNP, symptoms of the genital organs, and sexual dysfunction was based on patients’ self-report using assessments which were compiled from standard PNP (EORTC QLQ-CIPN20) and sexuality (IIEF-5, FSFI-d, EORTC SHQ-C22) questionnaires as well as from newly generated questions for symptoms of the genital organs. The survey had a pilot character. Since there was no questionnaire covering the three topics PNP, symptoms of the genital organs, and sexual dysfunction, and since compliance of the participants had to be ensured, only parts of existing questionnaires were used. For organizational reasons as well as due to the COVID-19 pandemic, clinical neurologic PNP validation or nerve function measurement were not performed additionally. 

## Results

Of the ninety-three persons contacted, n=31 (33%) patients participated in the survey. N=21 (68%) completed the questionnaires. Two women had to be excluded because their PNP could not be related temporally to chemotherapy due to the onset of PNP fourteen years and one year after the end of chemotherapy, respectively. Two men were excluded because they were not receiving chemotherapy or immunotherapy and thus could not have PNP symptoms of the genital organs due to these therapies. Consequently, n=17 (55%) evaluable questionnaires (four men (Table 1 (Tab. 1)) and thirteen women (Table 2 (Tab. 2)) were available. 

Three (75%) of the four men with PNP reported sensitive disorders of the genital organs (Table 3 (Tab. 3)). The penis and glans were most commonly affected. Three (75%) men had various degrees of severity of erectile dysfunction and one (25%) man reported reduced libido. The most severe form of symptomatology was reported by three (75%) men for PNP, by one (25%) for genital organ symptoms as hypersensitivity in penis and glans, and by two (50%) for sexual dysfunction. All men had previously received chemotherapy, and one patient had additionally received previous immunotherapy.

Sensitive symptoms of the genital organs were reported by nine (69%) of thirteen women (Table 4 (Tab. 4)), most severely as pain and hypersensitivity in the vagina, but also in the clitoris and labia. The most severe form of symptomatology was reported by two (15%) women for PNP and by two (15%) for genital organ symptoms. Twelve (92%) women reported reduced libido. Five (38%) women were sexually inactive during the four weeks prior to the survey. Four (80%) of them reported genital organ symptoms. All of the eight sexually active women reported arousal, lubrication, and orgasm disorders, and five (63%) reported genital organ symptoms. The most severe sexual dysfunction was lubrication disorder. No sexually active woman and two of the five (40%) sexually inactive women reported the most severe form of symptomatology of the genital organs. 

Seven (54%) of the thirteen women had received anti-hormone therapy (AHT) previously and/or did so at the time of the study. Three (50%) of the six women with previous AHT and six (60%) of the five women on AHT at the time of the study reported sensitive symptoms of the genital organs, whereas five (83%) of the six women without AHT reported these symptoms. Eight (89%) of the nine women with sensitive symptoms of the genital organs had received chemotherapy previously and/or did so at the time of the study, and one (11%) had undergone previous immunotherapy as well as receiving it at the time of the study. Seven (54%) of the thirteen women were under fifty-two and thus defined as premenopausal, whereas six (46%) women were fifty-two or older and defined as postmenopausal. Premenopausal women were more likely to report sexual activity (pre=5 (71%) vs. post=3 (50%)) and sensitivity symptoms (pre=5 (71%) vs. post=4 (67%)) after chemotherapy or immunotherapy. Five (71%) of the premenopausal women were non-overweight and two (29%) were overweight. Among postmenopausal women, five (83%) were non-overweight and one (17%) was overweight. Of the ten non-overweight women, six (60%) were sexually active and seven (70%) had symptoms of the genital organs. Two (67%) of the three overweight women were sexually active and had symptoms of the genital organs.

## Discussion

The aim of the study was to investigate the potential association between PNP, sexual dysfunction, and physical activity behavior. The main findings can be summarized as follows: Our limited data may indicate sensory symptoms of the genital organs in patients undergoing chemotherapy and immunotherapy. This is supported by the reported symptoms of the skin cancer patient who received immunotherapy and no chemotherapy. This kind of genital organ symptomatology does not seem to be directly related to sexual dysfunction. Nevertheless, the association between PNP and symptoms of the genital organs seems to be much more pronounced in sexually inactive women. However, due to the small number of subjects, no correlation analysis but descriptive statistics were performed.

PNP is caused by the destruction of nerve fibers due to chemotherapy and immunotherapy [1]. Chemotherapy and immunotherapy may also cause damage to sensory and autonomic nerve fibers of the genital organs, which could be causal for sensory symptoms and sexual dysfunction. Chemotherapy in premenopausal age can lead to premature ovarian failure [9]. This decline in estrogen production typically manifests itself in the form of menopausal symptoms, which are physical changes, such as hot flashes, urogenital problems, or vaginal changes. Menopause is accompanied by declining testosterone levels, which are associated with reduced libido, sexual arousal, and vaginal lubrication [10]. These chemotherapy-induced hormonal changes may also be a reason for sensory symptomatology. It remains unclear whether the sensory symptoms were caused by the destruction of the ovarian function through chemotherapy or by PNP of the genital organs induced by chemotherapy. Since men also develop sensory symptoms, this would support the theory of PNP of the genital organs. AHT may also trigger sexual dysfunction due to the sudden deprivation of estrogen and the associated disruption of hormonal balance [11]. Data on hormonal changes in the patients surveyed before cancer treatment compared to during or after were not available. The ethics votes did not take into account blood sampling (sampling of hormone status). Future studies must take this into account. Consequently, no statement can be made about the influence of possible hormonal changes in the patients studied. The data from the survey suggest that AHT does not appear to be the main cause of either sexual dysfunction or genital sensory symptoms. When interpreting the results, it should be taken into account that the sexually inactive women were eight years older than the sexually active women, with an average age of fifty-seven years, since aging is associated with reduced sexual activity due to physiological changes [12], [13]. 

The small number of cases is a limitation of the study, since it reduces the significance of the results. Moreover, the menopausal status of the women before therapy onset was not recorded. In addition, the effect of sexual activity on men could not be investigated, as all patients were sexually active in the last four weeks. Another limitation was that not all questions of the FSFI and the EORTC questionnaires were used, but only a selection. Future surveys should include complete questionnaires or subcategories of such questionnaires. A strength of the study is that this is the first investigation of this kind in cancer patients. Thus, sexual dysfunction, sensory symptoms of the genital organs, and PNP in cancer therapy could now be better understood in the context of each other. Future studies need to include more patients and measure physical activity behavior of patients to assess and analyse an association between physical activity level and occurrence of PNP symptomatology as well as sexual dysfunction. From this, correlation analyses can be generated, and physical activity therapy prevention and rehabilitation programs can follow. Exercise therapy can improve PNP through sensorimotor exercise and whole-body vibration exercise, and sexual dysfunction can be improved through pelvic floor muscle exercise [14], [15], [16].

## Conclusion

Our limited data suggest the possible existence of genital organ sensitive symptoms in chemotherapy and immunotherapy patients. Larger-scale studies are needed that can assess the influence of cancer therapy-induced PNP, physical activity level, and hormonal balance on genital organ sensitive symptoms and sexual dysfunction, which is necessary to be able to narrow down the reasons for the present observations. Our study addresses the problem that response rates to surveys on sexual difficulties and dysfunction are often low [17], which needs to be considered when planning further studies. Response rates appear to be higher when surveys are conducted in person [17].

## Notes

### Patient consent

All patients gave informed consent to participate.

### Ethics statement

The ethics votes of the IST University (PNPGB20200620) and the University Hospital Cologne (17-165) are available.

### Competing interests

The authors declare that they have no competing interests. Sections of this publication are part of Dirk Brodesser’s master thesis.

## Figures and Tables

**Table 1:**
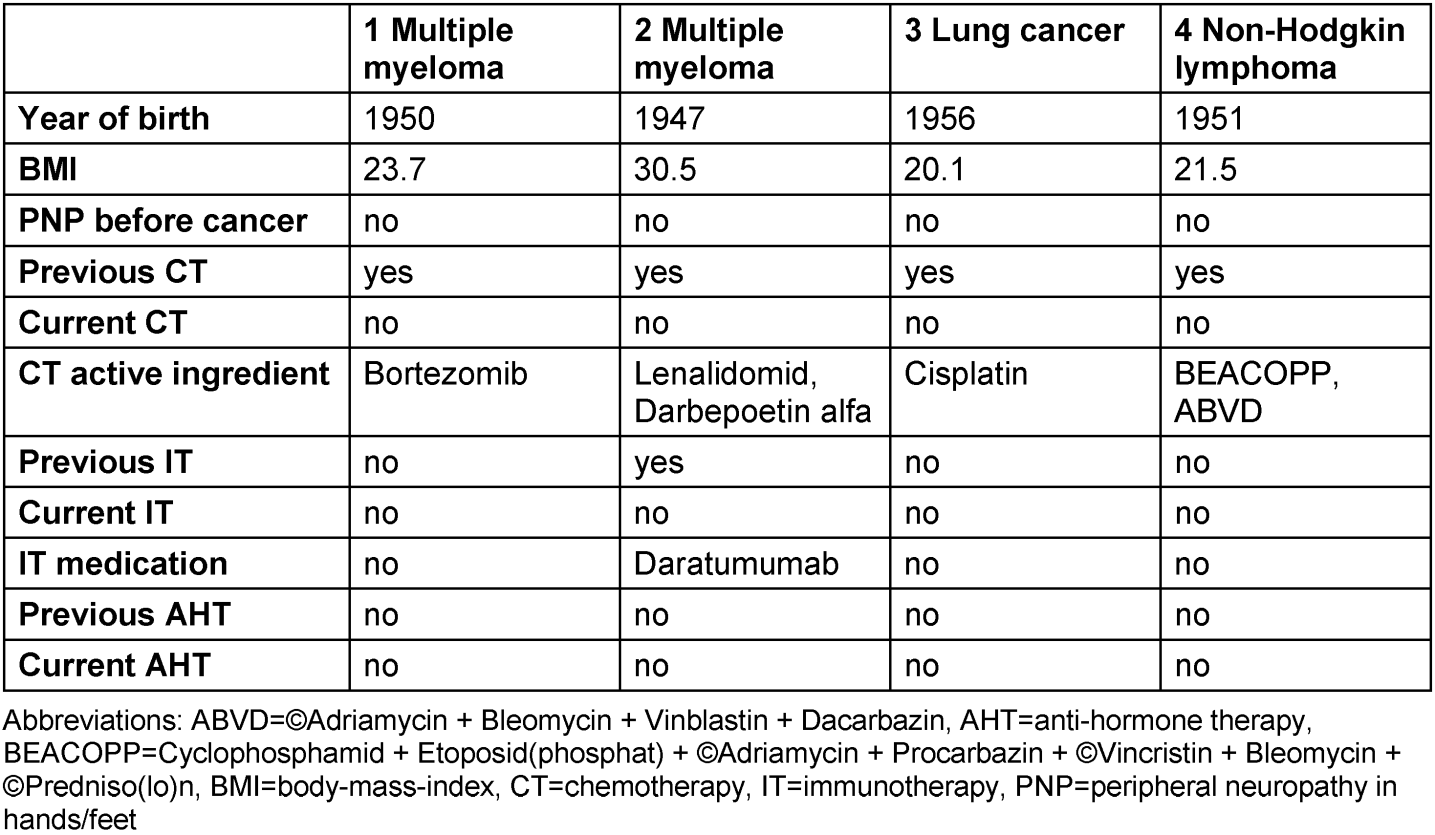
Overview of the treatments of and side effects in men

**Table 2:**
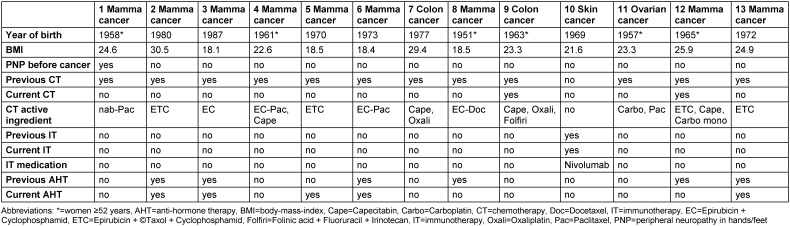
Overview of the treatments of and side effects in women

**Table 3:**
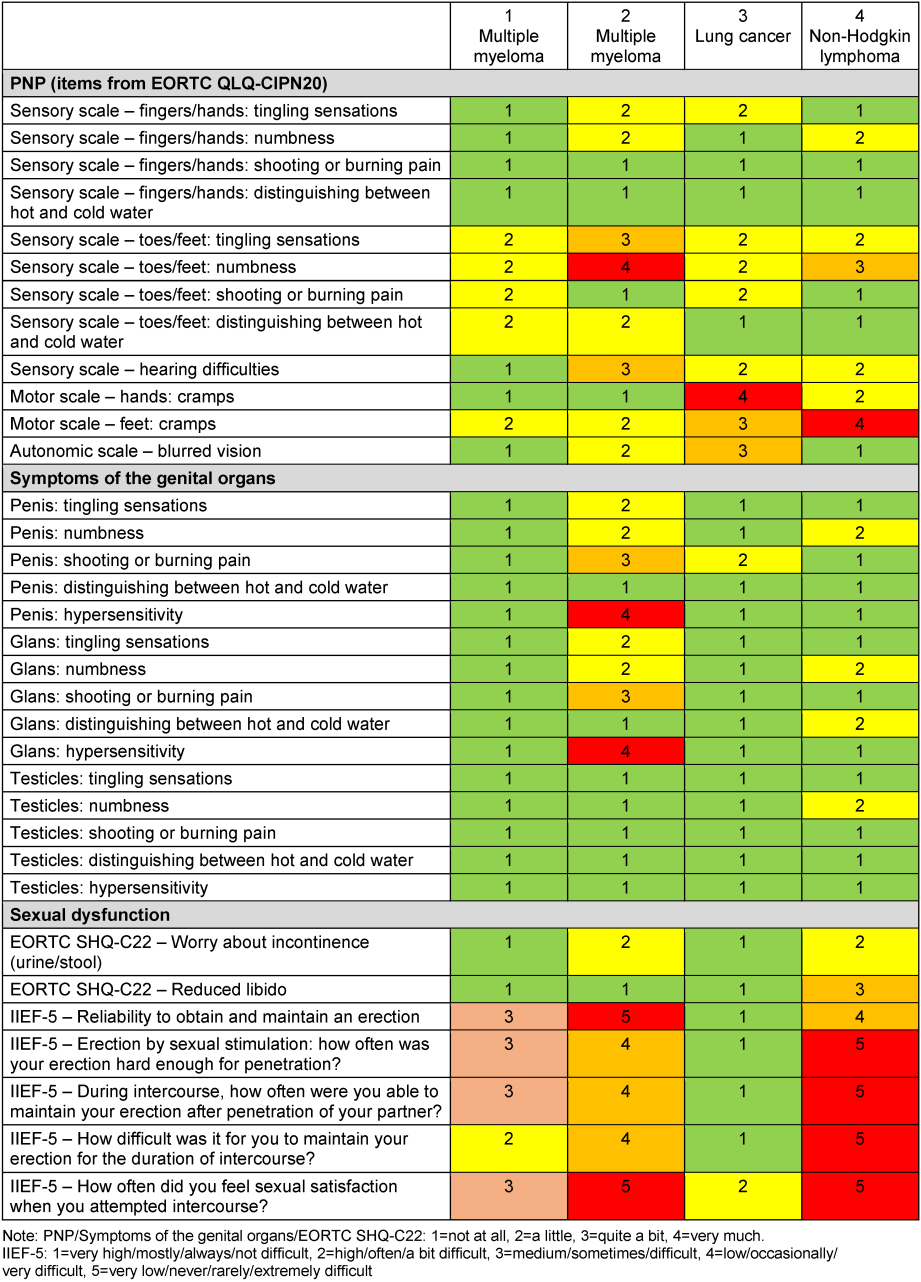
Overview of the extent of PNP symptoms, senstive symptoms of the genital organs, and sexual dysfunction in men

**Table 4:**
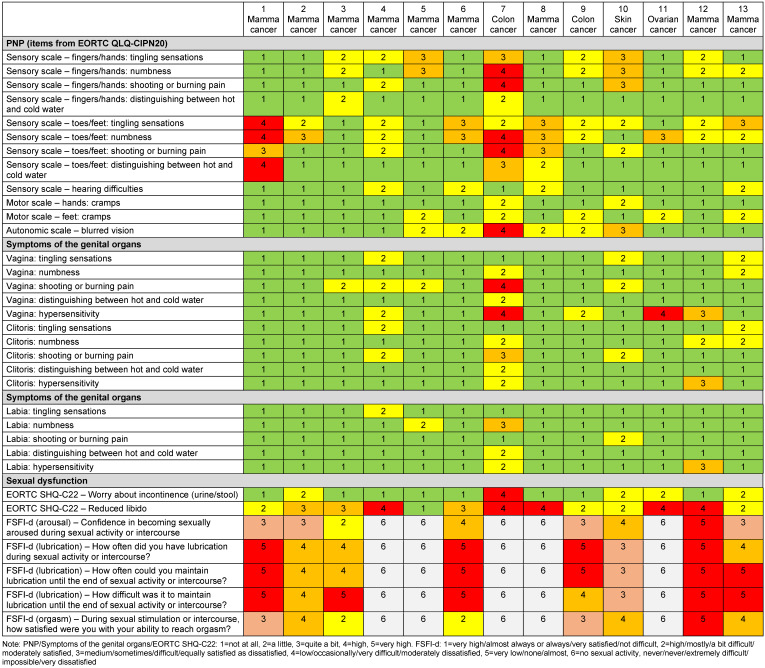
Overview of the extent of PNP symptoms, sensitive symptoms of the genital organs, and sexual dysfunction in women
